# Fully automated segmentation of left atrium and pulmonary veins in late gadolinium enhanced MRI

**DOI:** 10.1186/1532-429X-18-S1-O84

**Published:** 2016-01-27

**Authors:** Qian Tao, Rahil Shahzad, Esra Gucuk Ipek, Floris F Berendsen, Saman Nazarian, Rob J van der Geest

**Affiliations:** 1 grid.10419.3d0000000089452978Radiology, Leiden University Medical Center, Leiden, Netherlands; 2 grid.21107.350000000121719311Division of Cardiology, Johns Hopkins University School of Medicine, Baltimore, MD USA

## Background

Late gadolinium enhanced (LGE) MRI enables scar assessment in the left atrial (LA) wall, providing valuable information for treatment planning and image based procedure guidance in patient with atrial fibrillation (AF). Accurate and objective segmentation of the anatomy of the LA and pulmonary veins (PV) is an important prerequisite for atrial scar assessment. However, segmentation of the LA and PV's from LGE MRI is highly complex due to morphologic variations and limited image contrast. The study aims to develop a fully automated method for LA and PV endocardial wall segmentation from LGE MRI.

## Methods

**Figure 1:**
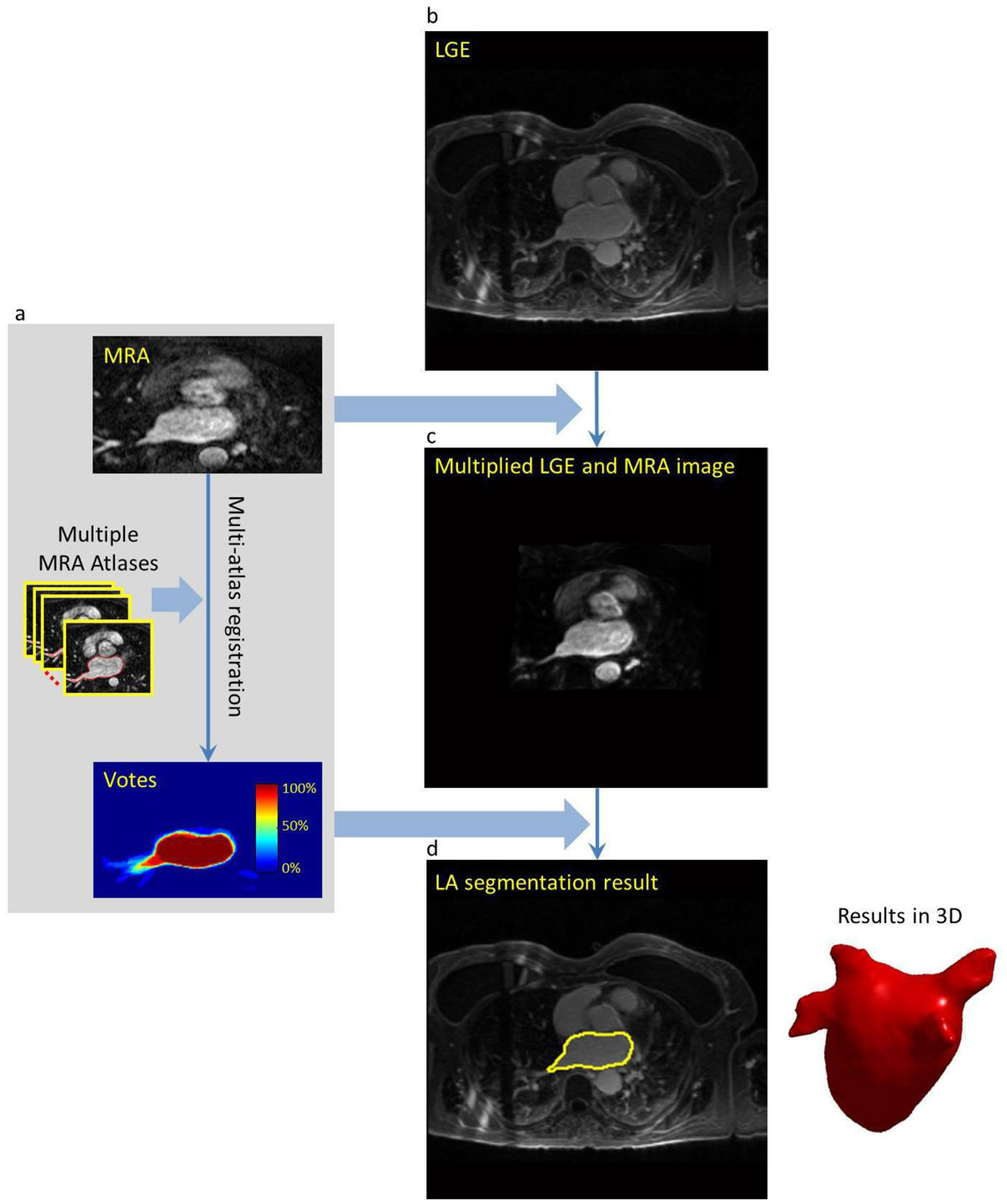
**Diagram of the proposed LA and PV segmentation method based on LGE and MRA sequences**.

Forty-eight AF patients (age 62 ± 7, 12 female) who underwent cardiac MRI before and after RF ablation therapy were included. A contrast-enhanced MRA sequence was used for assessment of the anatomy followed by LGE MRI for atrial scar assessment. A multi-atlas based segmentation approach was used to first segment the MRA: 10 manually annotated MRA atlases were non-rigidly registered to the MRA sequence, providing a majority-voted LA segmentation (Figure 1a). The MRA was subsequently registered to the LGE sequence to generate a multiplied LGE-MRA volume with improved contrast (Figure 1c). Three-dimensional region-growing and active contour refinement was applied to the multiplied volume for refined segmentation of the LA endocardial wall and further identification of PVs (Figure 1d). The automated segmentation results were compared to manual segmentation on LGE MRI from an experienced observer. The segmentation accuracy, i.e. surface-to-surface distance and Dice overlap index, was evaluated for LA and PV regions separately.

## Results

**Figure 2:**
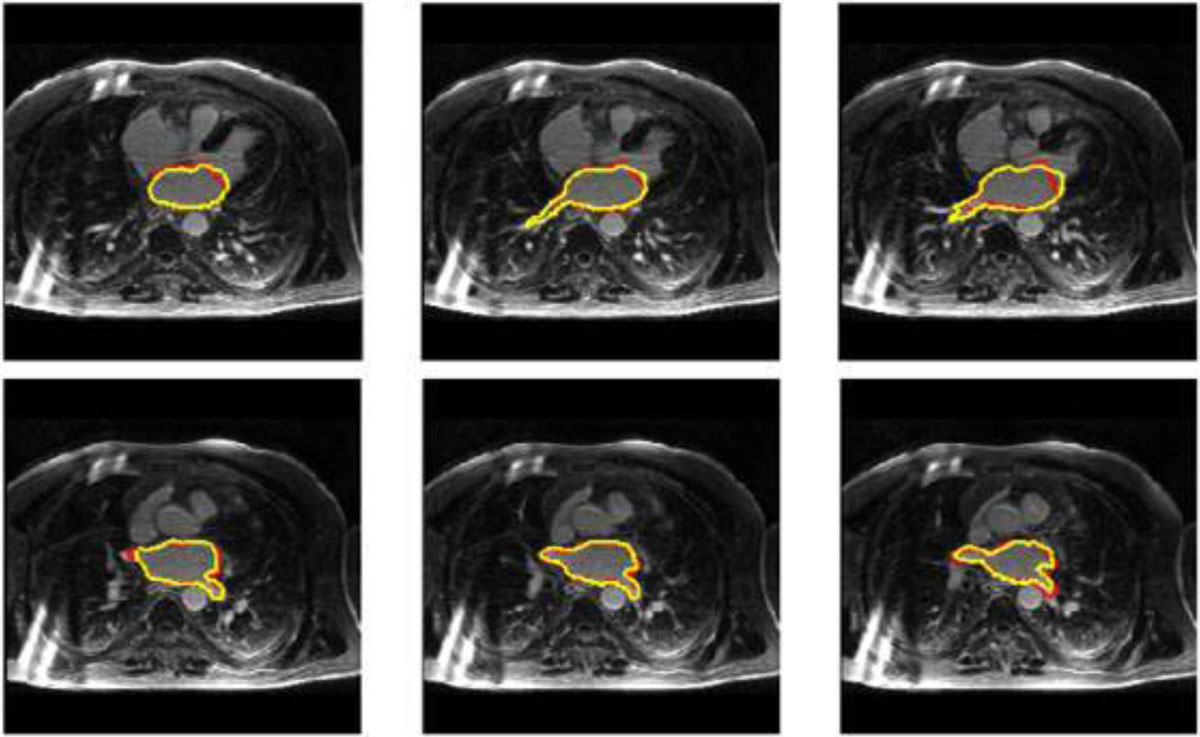
**Example of LA and PV segmentation results in a subject**. Red line: manual segmentation. Yellow line: automated segmentation.

The LA and PV ostia were fully and automatically segmented in all participants. Figure 2 shows an example of the automated segmentation results. In comparison to the manual LGE segmentation, the method that combines both MRA and LGE sequences yielded a surface-to-surface distance of 1.53 ± 0.48 mm in the LA region, and 2.28 ± 0.76 mm in the PV region. The Dice overlap index between the automated and manual segmentation was 83% ± 6% in the LA region and 74% ± 12% in the PV region.

## Conclusions

Automated segmentation of endocardial LA and PV wall is feasible by the multi-atlas approach combined with region growing and active contour methods, utilizing both LGE and MRA sequences. With future work focusing on epicardial wall identification, the proposed method may improve the reproducibility of atrial scar analysis for AF patients.

